# Supplementary material for: A Combined Differential Proteome and Transcriptome Profiling of Fast- and Slow-Twitch Skeletal Muscle in Pigs
Source: Foods. 2022 Sep 14;11(18):2842. doi: 10.3390/foods11182842 (PMC9497725; doi:10.3390/foods11182842)
Supplement: Supplementary file 1 [file foods-11-02842-s001.zip › Supplementary materials/Supplementary materials caption.pdf]

Supplementary Figures

Figure S1. Experimental design and workflow for quantitative proteomic difference analysis between *Biceps femoris* (Bf) and *Soleus* (Sol).

Figure S2. Summary of iTRAQ-based proteomics data for porcine skeletal muscles. Basic information statistics for spectra, peptide and protein. (B) Distribution of peptide length. (C) Distribution of protein mass. (D) Distribution of peptide sequence coverage. (E) Distribution of proteins containing different number of identified peptides.

Figure S3. Heatmap of DAPs between fast-twitch and slow-twitch muscles. Protein expression were log_{10} transformed and displayed in colors from red (high abundance) to blue (low abundance). Columns are clustered using Pearson's correlation distance. The samples are classified into two clusters, the first cluster comprised Bf28, Bf35 and Bf36, and the second cluster included Sol28, Sol35 and Sol36.

Figure S4. Validation of DAPs and correlation analysis. (A) Validation of DAPs using qRT-PCR (n = 3). Relative mRNA expression levels were calculated using the $2^{-\triangle\triangle ct}$ value method, and porcine *HPRT* was used as an endogenous reference gene. The X-axis represents DAPs and Y-axis represents the log_2 (fold change) of DAPs derived from qRT-PCR and iTRAQ data. (B) Correlation analysis of the expression of DAPs between qRT-PCR and iTRAQ data. The X and Y-axis represent the log_2 (fold change) of DAPs detected by qRT-PCR and iTRAQ data, respectively.

Table S1. Primers used for qRT-PCR analysis.

Table S2. Proteins identified in porcine skeletal muscles.

Table S3. Function annotation of all identified proteins in this study.

Table S4. Detailed information of DAPs between fast-twitch and slow-twitch muscles.

Table S5. GO and KEGG enrichment analysis of DAPs.

Table S6. Annotation information of nine quadrant associate analysis of mRNA and proteins.
