# Supplementary material for: A Combined Differential Proteome and Transcriptome Profiling of Fast- and Slow-Twitch Skeletal Muscle in Pigs
Source: Foods. 2022 Sep 14;11(18):2842. doi: 10.3390/foods11182842 (PMC9497725; doi:10.3390/foods11182842)
Supplement: Supplementary file 1 [file foods-11-02842-s001.zip › Supplementary materials/Table S1. Primers used for qRT-PCR analysis.pdf]

Table S1 Primers used for qRT-PCR analysis.

Gene name	Forward primer	Reverse primer
PVALB	TTCTCCCCAGATGCCAGAGA	GGAGAATTCGTCAGCCCCAA
DNAJC3	TCAGAGACGGCAGATACACAG	AGCTTCCACAGGCTTCTCATC
ATP2A1	CCTCTGCCGATGATCTTCAA	ACACGGTTCAAAGAGGTGGA
COL1A2	CTGGTCTTGGCGGGAACTTT	AGGACCAGTCTGACCAGGTT
REEP5	TACATGCTGAAGTGCGGCTT	TCCTTAGTGATGGCATCCGC
LGALS1	TCGCCAGCAACCTGAATCTCAA	CACACCTCCACGACACTTCCA
CA3	TGGAGTGGCTGTAGTTGGCATT	GGCAGGATGGGTTGAAGTTCGT
MYOZ2	GCAGAGGAAACAGCAAGCATCA	TGGCACCACGGTTACTGAGG
MYH7	GCCGAGAAGGATGAGGAGATGG	ATGCGATTGGCGTGGCTGAG
CSRP3	AGCACGGACACAGGCGAACA	GCCACCTCCCATCACCTTCTCA
Tnnc1	CGAGGTGGATGAAGACGGGAGT	CGCCTGTCGCCTGAAGCATT
HPRT	GTCAACGGGCGATATAAAAGT	GGGCATAGCCTACCACAAACT
