# Supplementary figures and images for: A Combined Differential Proteome and Transcriptome Profiling of Fast- and Slow-Twitch Skeletal Muscle in Pigs
Source: Foods. 2022 Sep 14;11(18):2842. doi: 10.3390/foods11182842 (PMC9497725; doi:10.3390/foods11182842)

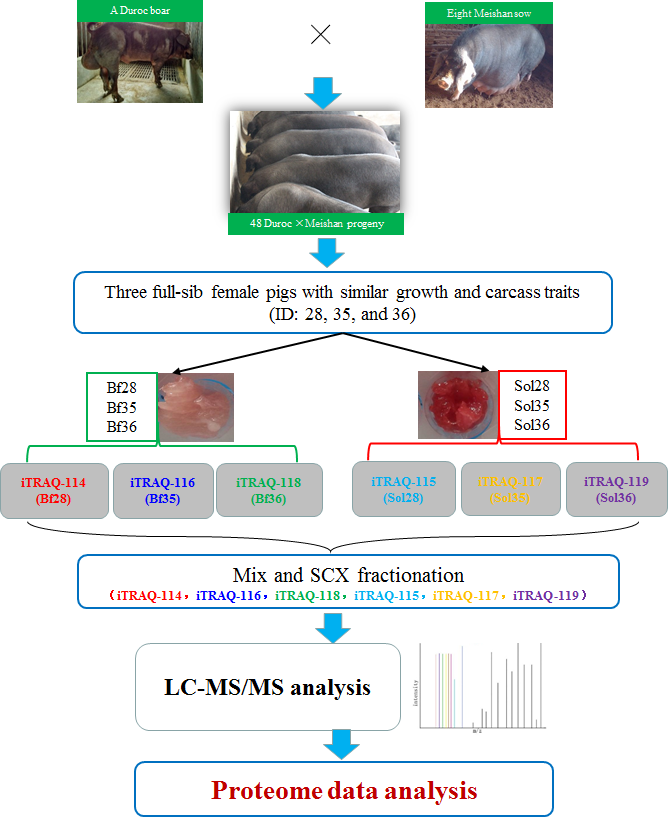

Supplement: Supplementary file 1 [file foods-11-02842-s001.zip › Supplementary materials/Figure S1. Experimental design and workflow for quantitative proteomic difference analysis between Biceps femoris (Bf) and Soleus (Sol).tif]

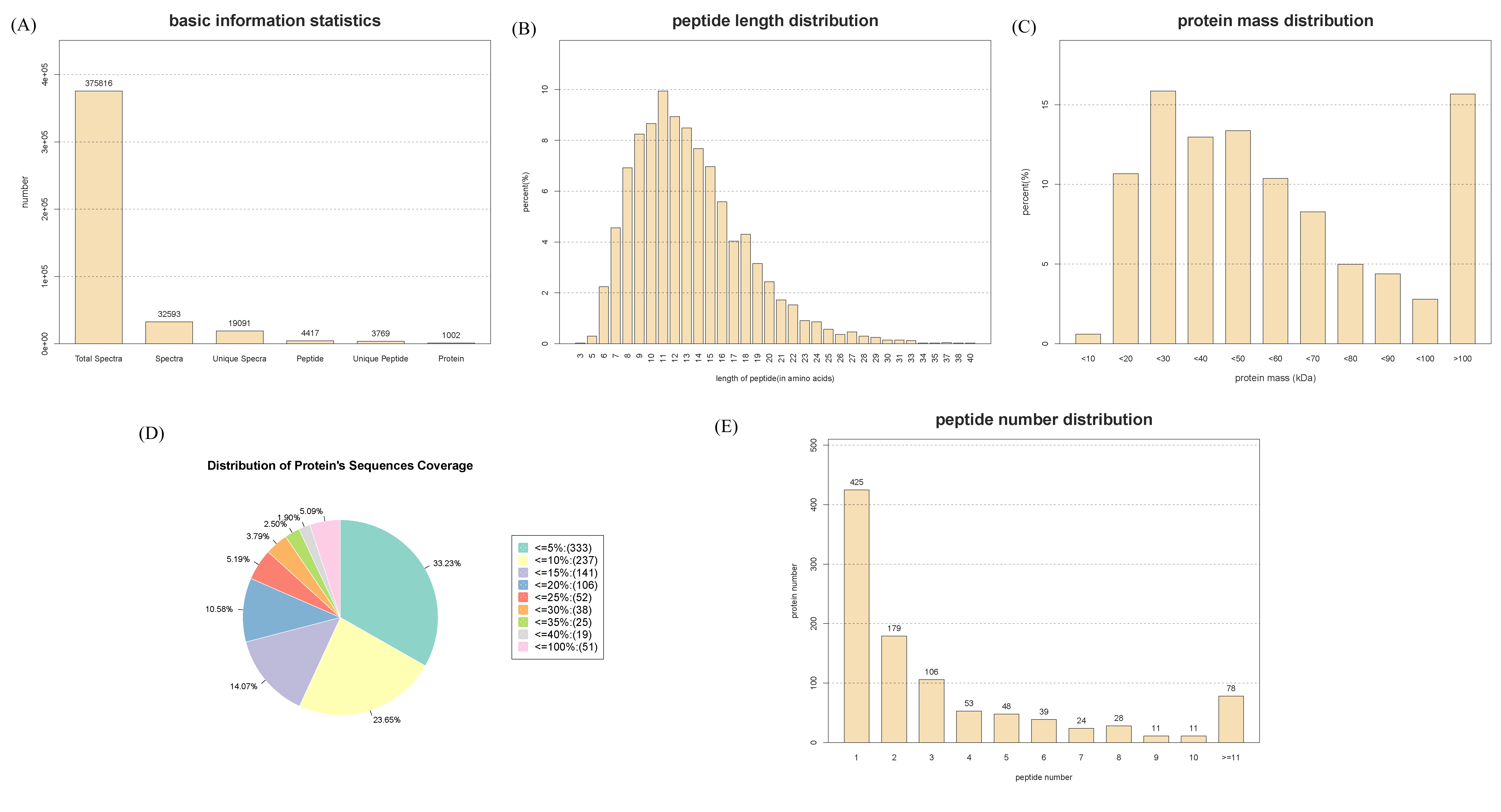

Supplement: Supplementary file 1 [file foods-11-02842-s001.zip › Supplementary materials/Figure S2. Summary of iTRAQ-based proteomics data for porcine skeletal muscles.tif]

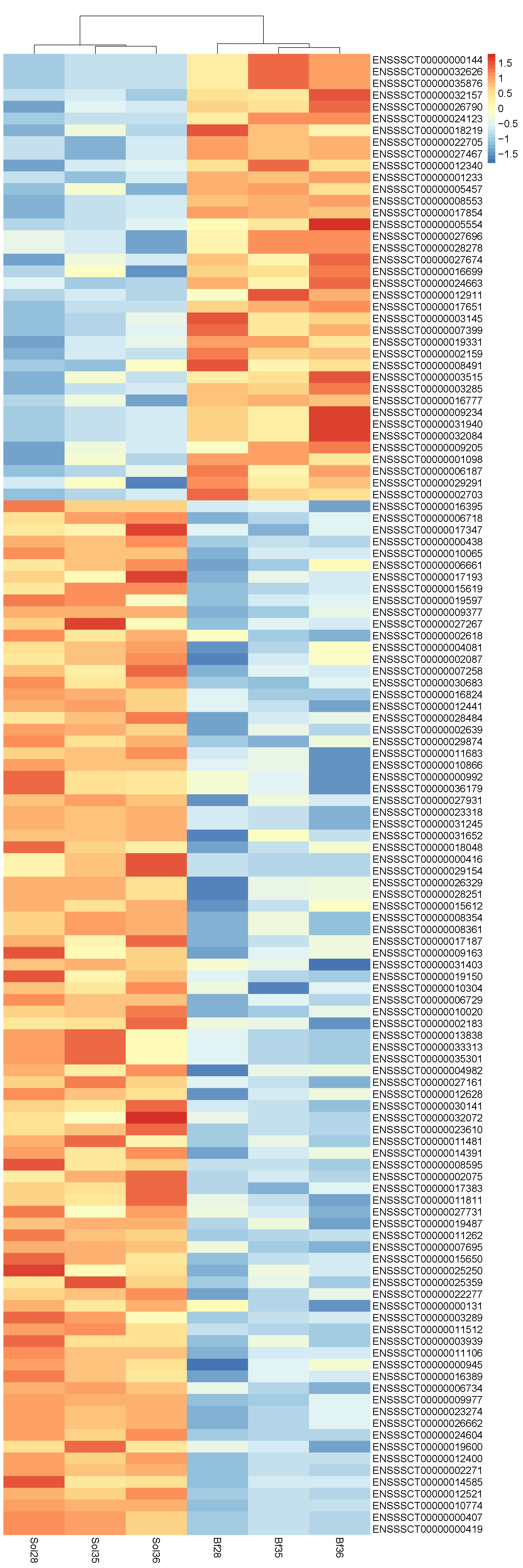

Supplement: Supplementary file 1 [file foods-11-02842-s001.zip › Supplementary materials/Figure S3. Heatmap of DAPs between fast-twitch and slow-twitch muscles.tif]

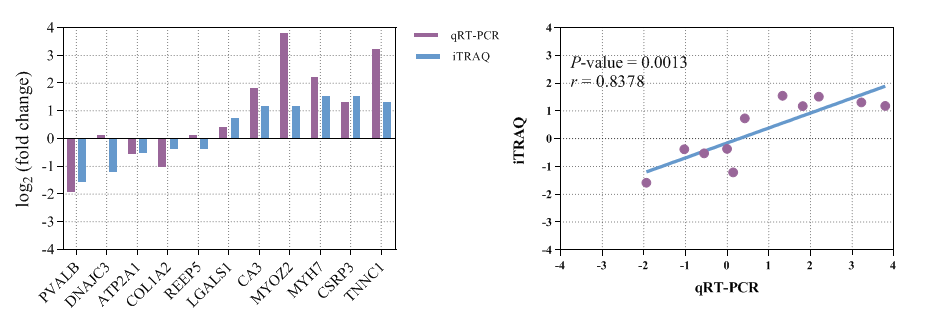

Supplement: Supplementary file 1 [file foods-11-02842-s001.zip › Supplementary materials/Figure S4. Validation of DAPs and correlation analysis.tif]
